# Temporal Changes in Functional and Structural Neuronal Activities in Auditory System in Non-Severe Blast-Induced Tinnitus

**DOI:** 10.3390/medicina59091683

**Published:** 2023-09-18

**Authors:** Ningning Shao, Maciej Skotak, Navya Pendyala, Jose Rodriguez, Arun Reddy Ravula, Kevin Pang, Venkatesan Perumal, Kakulavarapu V. Rama Rao, Namas Chandra

**Affiliations:** 1 Center for Injury Biomechanics, Materials and Medicine, Department of Biomedical Engineering, New Jersey Institute of Technology, 111 Lock Street, Newark, NJ 07102, USA; 2 NeuroBehavioral Research Laboratory, VA New Jersey Health Care System, Research and Development (Mailstop 15), 385 Tremont Ave, East Orange, NJ 07018, USA; 3 Department of Pharmacology, Physiology and Neuroscience, Rutgers-New Jersey Medical School, Newark, NJ 07103, USA

**Keywords:** hearing loss, blast overpressure, impulsive noise, tinnitus

## Abstract

*Background and Objectives*: Epidemiological data indicate that blast exposure is the most common morbidity responsible for mild TBI among Service Members (SMs) during recent military operations. Blast-induced tinnitus is a comorbidity frequently reported by veterans, and despite its wide prevalence, it is also one of the least understood. Tinnitus arising from blast exposure is usually associated with direct structural damage that results in a conductive and sensorineural impairment in the auditory system. Tinnitus is also believed to be initiated by abnormal neuronal activities and temporal changes in neuroplasticity. Clinically, it is observed that tinnitus is frequently accompanied by sleep disruption as well as increased anxiety. In this study, we elucidated some of the mechanistic aspects of sensorineural injury caused by exposure to both shock waves and impulsive noise. The isolated conductive auditory damage hypothesis was minimized by employing an animal model wherein both ears were protected. *Materials and Methods*: After the exposure, the animals’ hearing circuitry status was evaluated via acoustic startle response (ASR) to distinguish between hearing loss and tinnitus. We also compared the blast-induced tinnitus against the well-established sodium salicylate-induced tinnitus model as the positive control. The state of the sensorineural auditory system was evaluated by auditory brainstem response (ABR), and this test helped examine the neuronal circuits between the cochlea and inferior colliculus. We then further evaluated the role of the excitatory and inhibitory neurotransmitter receptors and neuronal synapses in the auditory cortex (AC) injury after blast exposure. *Results*: We observed sustained elevated ABR thresholds in animals exposed to blast shock waves, while only transient ABR threshold shifts were observed in the impulsive noise group solely at the acute time point. These changes were in concert with the increased expression of ribbon synapses, which is suggestive of neuroinflammation and cellular energy metabolic disorder. It was also found that the onset of tinnitus was accompanied by anxiety, depression-like symptoms, and altered sleep patterns. By comparing the effects of shock wave exposure and impulsive noise exposure, we unveiled that the shock wave exerted more significant effects on tinnitus induction and sensorineural impairments when compared to impulsive noise. *Conclusions*: In this study, we systematically studied the auditory system structural and functional changes after blast injury, providing more significant insights into the pathophysiology of blast-induced tinnitus.

## 1. Introduction

Blast injuries have become one of the most debilitating traumas among veterans, active-duty Service Members (SMs) [1], and recently even civilians due to the increasing frequency of terror attacks worldwide [2]. According to data from the Armed Forces Health Surveillance Center (AFHSC) in 2013, 76% to 83% of the reported injuries were classified as mild TBI (mTBI). Unlike blunt force trauma, the hallmark of blast injury is diffuse (“invisible”) neurotrauma, which is widespread in various brain regions. This diffuse neurotrauma arises from compression and tension applied to the tissues as the shock wave transverses through the brain parenchyma without permanently injuring the external skull. Among many blast-induced disabilities, auditory concerns, including hearing loss and tinnitus, are the most reported cases among veterans, SMs [3], and the civilian population [4,5]. Hearing loss is the failure to process specific sound frequencies due to conductive damage and/or sensorineural damage to the auditory system. Tinnitus is the perception of sound that persists in the absence of an external acoustic source [6]. The analysis of hearing threshold shifts and self-reported otologic complaints from a sample of over 250 patients with blast-related injuries demonstrated that 49% of the subjects had experienced tinnitus. In general, the hearing threshold shifts after deployment compared with pre-deployment were prominent, indicating significant changes in the patient’s hearing [1].

Additionally, hearing loss and tinnitus have been shown to cause sleep deprivation, difficulty in concentrating and socializing, and the development of suicidal tendencies [3], all of which highly decrease the quality of life. Despite years of research, there still exists a knowledge gap in identifying the most prominent mechanisms behind blast-induced tinnitus. Therefore, the development of safe and effective treatment is precluded, contributing to the dramatic increase in the cost of long-term care for these patients with blast-induced hearing impairment [7]. Ethical concerns make mechanistic human studies challenging, while it has been suggested that animal models provide alternative research platform and thus are critical to elucidate underlying mechanisms [8].

The mounting experimental evidence suggests that exposure to blasts can not only cause conductive damage but also neurotrauma. Conductive abnormalities in the peripheral auditory system (PAS), including tympanic membrane (TM) rupture and ossicular chain disruption, are well characterized and understood [9,10,11]. These conditions can be wholly or partially reversed within a few months after injury either spontaneously or with surgical interventions. However, sensorineural hearing deficits have more permanent lasting effects among the affected population, and there are only limited options for safe and effective treatment. In this context, the need to investigate sensorineural damage in the central auditory system (CAS) is important and urgent. The obvious choice to address that need and expand our knowledge about the mechanisms responsible for tinnitus is to use animal models exposed to blast (or impulsive noise) in controlled conditions. Previous animal studies of blast-induced auditory problems discovered changes lasting up to 3 months in the auditory cortex (AC) and dorsal cochlear nucleus (DCN) after exposure to blast with 152 kPa peak overpressure [12,13,14]. Exposure to a shock wave with 97 kPa peak overpressure resulting in hyperactivity along the pathway of CAS was observed using manganese-enhanced magnetic resonance imaging (MEMRI) after five weeks [15]. Additionally, at the cellular level, astrocyte activation and axonal degeneration were observed [12,13,16].

Tinnitus is also one of the most common disabilities among veterans returning from war zones, considering they have been typically exposed to multiple blast episodes. To date, some of the chief causes of tinnitus include occupational continuous noise exposure, blast exposure, aging, and ototoxic drugs [17]. Tinnitus is often accompanied by hearing loss. The ototoxicity of certain medications, such as sodium salicylate, have been adapted for research purposes, and this kind of animal model has been widely utilized to study mechanisms behind tinnitus [18,19,20,21]. Salicylate is an active ingredient or metabolite in many common drugs, such as aspirin. At a high dosage, it can temporarily induce moderate hearing loss and cause tinnitus in humans and animals. It has been demonstrated that, in the peripheral system, it acts as a competitive antagonist for the chloride anion binding site of prestin [22], the motor protein of outer hair cells (OHCs), resulting in the inhibition of OHC motility. Changes in the spontaneous firing rate in the auditory nerve have also been observed [23]. In the CAS, salicylate alters neurotransmitter homeostasis, i.e., increasing the release of γ-aminobutyric acid (GABA), which then inhibits further neurotransmission as compensation for increased neural traffic. It can also cause spontaneous firing in the CAS and, in some cases, in the limbic system [20,24]. 

In this study, we used the sodium salicylate-induced tinnitus (SSIT) model as a positive control and compared the results to those observed in the blast exposure model. Our main goal was to test the hypothesis that a synergistic effect exists associated with simultaneous exposure to impulsive noise and shock wave greater than the effect of each individual exposure, as these conditions represent typical military training and combat environments. The progress of the injury with respect to time and location along the auditory pathway should also guide the timing and sites of target treatments. Examination of the two separate injury modes (impulsive noise and shock wave, respectively) indicates that the development of all-inclusive personal protection equipment (PPE) may not be feasible. 

## 2. Materials and Methods

### 2.1. Animals 

Adult 10-week-old male Sprague Dawley (Charles River Laboratories) rats weighing 350 ± 50 g were used in all the studies. The animals were housed with free access to food and water in a 12 h dark–light cycle at 22 °C. All experimental procedures followed the guidelines of Care and Use of Laboratory Animals approved by Rutgers University Institutional Animal Care and Use Committee before experiments. Rats were divided into three groups: (1) sham, (2) animals exposed to shock wave with 180 kPa peak overpressure (BLAST), and (3) sodium salicylate-induced tinnitus (SSIT) group. Animals in the sham group were placed outside of the shock tube exposed to impulsive noise with 90 kPa (193 dB) intensity without experiencing the shock wave. All animals’ ears were protected by Mack’s^®^ silicone earplugs (Warren, MI, USA).

A group of 18 rats was used in the following functional tests for each group: (1) acoustic startle response, (2) auditory brainstem response, (3) behavior tests. However, one animal in the blast group and three animals in the sham group died due to failure of recovery after anesthesia.

Another group of 15 animals were used for immunohistochemistry: 5 animals in the sham group, 10 animals that were blast injured at post-injury day 1 (PID-1, *n* = 5), and PID-7 (*n* = 5). For immunofluorescence studies, each brain tissue was processed to obtain several sections (>10) from the auditory cortex. Each section was used for the identification of neurotransmitter receptors in neurons by double immunofluorescence analysis. 

### 2.2. Blast Exposure

Rats were exposed to a single shock wave at the New Jersey Institute of Technology (Center for Injury Biomechanics, Materials, and Medicine) in the shock tube, as described in previous publications [25,26,27]. Briefly, prior to blast exposure, all animals were randomly selected into three groups (sham, BLAST, SSIT) and were anesthetized with 5% isoflurane released in a chamber containing 95% air and 5% CO_2_ until rats were unresponsive to paw and tail pitch. The animals were then exposed to the shock wave with 180 kPa peak overpressure for approximately 6 ms (impulse of 330 kPa∙ms) [27,28].

### 2.3. Sodium Salicylate-Induced Tinnitus (SSIT)

The positive control group of animals with sodium salicylate-induced tinnitus was used to compare with the blast-induced tinnitus group. The sodium salicylate was delivered daily for 42 days via intraperitoneal injection at the dosage of 300 mg/kg (the minimum effective dose for rats is 150 mg/kg [21]). Functional recordings were conducted within 2 h post-injection to maintain the overdosing window, according to previous studies [20]. 

### 2.4. Acoustic Startle Response (ASR)

Overall, 18 animals were involved in this study. Before blasts, a prescreening procedure was implemented to set up the baseline of the normal startle response. Only animals with the GAP/PPI index within the range of standard deviation of the average were included for the following studies. All animals met this prescreening test in this study. Then, animals were divided into sham, blast, and sodium salicylate-induced groups. After each procedure, for both sham and blast groups, ASR was recorded at post-injury days 1, 7, 15, 28, and 42. For the sodium salicylate group, ASR was tested within 2 h after injection.

Seven frequency bands (6–8 kHz, 8–10 kHz, 10–12 kHz, 12–14 kHz, 14–16 kHz, 16–18 kHz, and 18–20 kHz) were used for frequency band response evaluation, and broadband noise (2–30 kHz) is chosen to probe the overall status of hearing. Alternatively, GAP detection can be used to inform about tinnitus because the phantom ringing sound, the signature symptom of tinnitus, can smear the detection of the gap in noise before the loud stimulus.

In clinical diagnostics, audiograms and surveys are used as evaluation tools. In animal studies, animal acoustic startle response (ASR) is used to diagnose hearing problems. Gap detection and pre-pulse inhibition (GAP/PPI) are well-established hearing response paradigms in rodents [14,29]. In general, an animal startles when subjected to a loud stimulus in the presence of normal background noise. However, when a slight gap in background noise is introduced before a loud stimulus, the startle response is greatly reduced. The counterpart to this is a period of silence followed by pre-pulse right before the loud stimulus, which also suppresses the startle response. The first paradigm is GAP detection, and the other is pre-pulse inhibition (PPI). PPI is believed to help identify partial hearing loss conditions. If an animal suffers partial hearing loss, it cannot detect the pre-pulse, which will not show reduction in startle response. By changing the pre-pulse frequency bands, we can pinpoint the partial hearing-loss frequency band and generate frequency loss mapping. 

GAP detection procedure. Background noise (delivered at 60 dB SPL (Sound Pressure Level)) consists of 2 kHz bandpass signals of 6–8, 8–10, 10–12,12–14, 14–16, 16–18,18–20 kHz bands, or a broadband noise (2–30 kHz). The startle stimulus, a 50 ms noise burst, was delivered at 115 dB SPL. A 40 ms silent period that began 90 ms before the startle stimulus served as the gap. Startle force was recorded in response to 3 conditions: (1) background noise alone, (2) the startle stimulus preceded by the silent gap, (3) the startle stimulus alone.

Pre-pulse Inhibition (PPI) procedure. PPI sound has the same frequency ranges compared to GAP sound, but no background noise was administered. Instead, the startle response was recorded in response to (1) the startle stimulus alone, (2) the startle stimulus preceded by a 40 ms, and 60 dB SPL acoustic pre-pulse beginning 90 ms before the startle stimulus. Acoustic pre-pulses consisted of the same bandpass signals used for background noise in the gap-detection procedure. All of the above sounds were synchronized with the piezo sensor so that we can initiate the recordings at the same time and distinguish the startle signals.

Both GAP and PPI procedures were run in tandem, a 5 min acclimatization period was provided at the beginning of the tests to acclimate the animals. Two trials of the startle stimulus without background noise were given after the acclimatization period to trigger and dispose of any initial exaggerated startle reflexes. The maximum amplitude of the reflexing response [30] was measured by Matlab; the amplitudes are recorded within 100 ms after the startle sound plays. The gap and prepulse inhibition index are defined as follows:

(1) $$GAP\;index=\frac{A\;gap}{A\;no\;gap}$$

(2) $$PPI\;index=\frac{A\;prepulse}{A\;no\;prepulse}$$

where (1) *A gap* is the amplitude of the startle response when the gap sound is played; (2) *A no gap* is the amplitude of the startle response when startle with background sound is played; (3) *A prepulse* indicates the amplitude of the startle response when *prepulse* sound is played; (4) *A no prepulse* indicates the amplitude of the startle response when startle without background sound is played.

### 2.5. Auditory Brainstem Response (ABR)

The integrity of the neuronal circuit between the cochlea and inferior colliculus was examined by auditory brainstem response (ABR) at post day 2, 10, 20, 30, and 42 in sham and BLAST groups. The SSIT group was examined within 2 h after injection. ABR thresholds were recorded as the parameter of functional evaluation of the neuronal circuit between the cochlea and inferior colliculus. For each above-mentioned time point, animals were examined in the tympanic membrane rupture conditions; TM-ruptured animals were excluded from the ABR examination. Then, animals were initially anesthetized. Three platinum-coated tungsten electrodes were inserted in the vertex, below the ipsilateral pinna, and in the hind leg muscles for the positive, negative and ground positions, respectively. Click and tone-burst stimuli at 4, 6, 8, 10, 12, 14, 16, 18, and 20 kHz were delivered through an TDT RZ6 open field speaker. Stimuli were played from 100 to 5 dB with a 5 dB stepwise decrease. ABR signals were amplified, band-filtered from 0.3 to 3 kHz, notch-filtered at 60 Hz, and averaged 300 times for click and tone-burst stimuli, respectively using the previously described equipment and software [31]. ABR threshold was defined as the lowest sound stimulus level at which ABR waves can be identified. 

### 2.6. Immunohistochemistry 

Rats were cardiac perfused with 4% paraformaldehyde in 9.6 g/L PBS. The heads of the animals were harvested, and the skin was removed to expose the dorsal surface of the skull. The cerebrum, cerebellum, and brain stem were exposed and harvested by breaking the occipital bone and parietal bone. The brain specimen was then fixed in 4% Paraformaldehyde in 9.6 g/L PBS for 2–4 days, rinsed in 9.6 g/L PBS, and stored in 30% sucrose 9.6 g/L PBS solution. Brains were then dissected into 20 μm thickness sections using a Rat Brain vibratome (Thermo Fisher Scientific, Waltham, MA, USA) and mounted on glass slides. The auditory cortex was identified by using “Rat Brain in Stereotaxic Coordinates” by Paxinos and Watson [32].

Tissue sections were fixed in ice-cold methanol (100%) solution for 10 min at −20 °C and blocked in 10% donkey serum at room temperature for 1 h in PBS containing 0.03% Triton X-100. Fixed tissues were incubated overnight at 4 °C with respective primary antibodies against NMDA-R1 (1:150, Abcam 68144), GABA A Receptor alpha 1 (1:500, Abcam 33299, GABRA1), NeuN (1:200, Abcam104224), CtBP2(1:300, Abcam128871), and Caspase 3(1:250, Abcam13847). NMDA-R1 and GABRA1 were double-stained with NeuN, respectively. Secondary antibodies conjugated with AlexaFluor 594 (red) were used for both NMDA and GABAA receptors separately, AlexaFluor 488 (green) was used for NeuN, and Anti fading reagent with DAPI (ProLong™ Gold Antifade Mountant with DAPI, Invitrogen, Waltham, MA, USA) was used before placing a cover slide.

### 2.7. Image Acquisition and Quantification 

Following immunostaining, after 12 h of drying in the dark room, slides were digitized (20× magnification) using Leica Aperio Versa 200 fluorescent microscope and slide scanner. Fluorescence intensities in the auditory cortex were quantitated using AreaQuant software Flv1 (Leica Biosystems, Danvers, MA, USA) and expressed as average fluorescence intensity/unit area.

### 2.8. Animal Behavioral Tests

#### Elevated Plus Maze (EPM)

EPM is an effective method to assess anxiety, preferred by many investigators as it relies solely on the natural behavior of the rat. It does not involve artificial cues (acoustic or temperature change), stressors (predator odor, foot shock), or any form of motivated or conditioned response (levers and food rewards). It evaluates the rat’s behavior by timing the preference to stay in the dark areas (increased anxiety) versus exploration of novel areas (normal behavior). On a test day, rats were transferred to a behavioral room and acclimated for 30 min. Rats were then placed in the center zone of the EPM facing toward the open arm, and recordings were initiated using ANYMAZE 7.1 software. The rat was allowed to explore for 5 min, which was recorded using the video camera controlled by ANYMAZE. The amount of time and distance spent in open arms versus closed areas were calculated as an indicator of anxiety.

### 2.9. Sleep Studies

Sleep studies were performed following the method described by using the PiezoSleep apparatus (Signal Solutions Inc., Lexington, KY, USA). Animal activities were recorded continuously in 24 h cycles. The animal movements were automatically recorded by sensors, filtered, and then processed. The frequency, amplitude, and peak spectral energy of the signal were evaluated in 2 s increments over a tapered 8 s window to automatically calculate sleep/wake decision statistics by simple linear discriminant analysis. Signals would be classified as sleep if they exhibited a periodicity with a consistent, low relative amplitude at a frequency in the typical breathing range (1–4 Hz). Wakefulness would be determined based on high amplitude signals with variable frequency and broad spectral energy. Signal features related to these properties were extracted and combined to obtain a decision statistic for each interval computed as the number of mean sleep bout durations. We extracted sleep/wake bout duration, percentage of time spent sleeping, and the hourly amount of sleep in a 24 h cycle and then compared results with control animals to identify sleep disturbances.

### 2.10. Statistical Analysis

Data are presented as mean ± standard error of the mean. Statistical significance is determined using mixed design factorial analysis of variance (ANOVA) to compare the data within sham, BLAST, and SSIT groups before and after blast with a post hoc analysis using paired-samples *t*-tests with Bonferroni correction to determine differences within groups. Normality and population variance homogeneity were assessed with Shapiro–Wilk and Levene’s tests, respectively. Repeated measure ANOVA was used for the statistical analysis of behavioral tests, with the following significance thresholds: * *p* < 0.05, ** *p* < 0.005, and *** *p* < 0.0005.

## 3. Results

### 3.1. Blast Alters Acoustic Startle Responses

**Figure 1:**
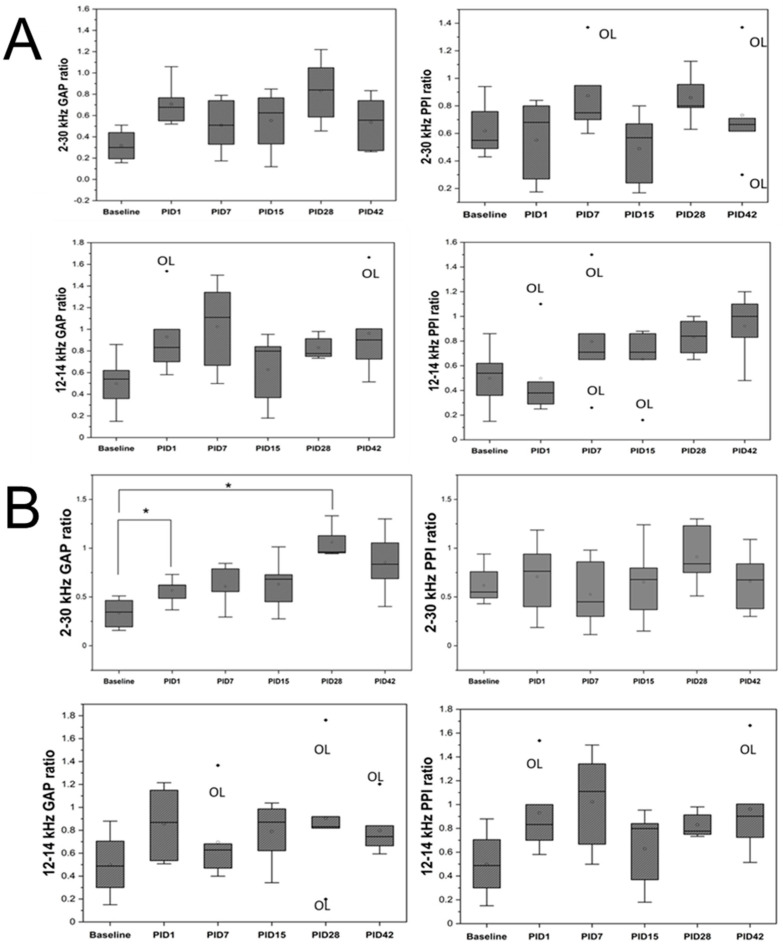

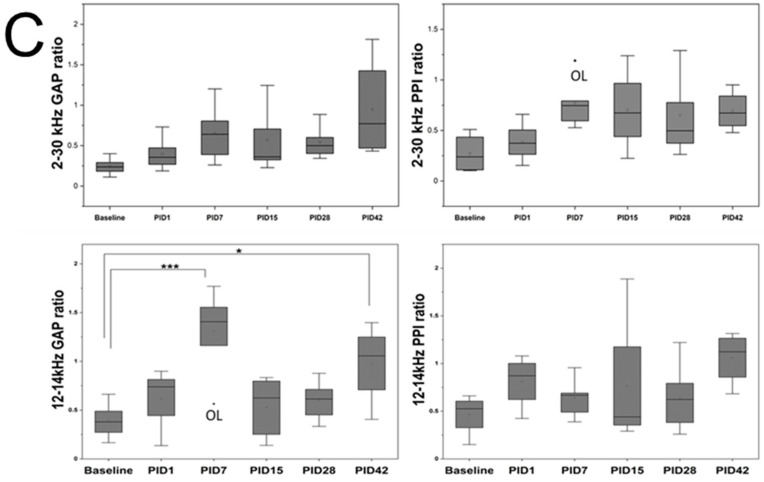
The results of acoustic startle response and PPI evaluation in: (**A**) in sham, (**B**) BLAST, and (**C**) SSIT cohorts. No statistically significant changes in GAP index nor PPI were noted for sham cohort (**A**). In the BLAST group, only changes in the GAP index on Day 1 and Day 28 were obvious (**B**). A significant change in the GAP index metric was also noted for the SSIT cohort on Day 7 and Day 28, but only at the 12–14 kHz band, not the broadband noise (2–30 kHz, (**C**)). OL, outliners. Repeated measure ANOVA was used for the statistical analysis of behavioral tests, with the following significance thresholds: * *p* < 0.05, and *** *p* < 0.0005.

The results of the ASR and PPI temporal screening in the sham, blast, and SSIT cohorts are presented in Figure 1. As shown in Figure 1B, in the broadband (2–30 kHz) range of the blast group, we observed a significant effect at the two time points: PID1 and PID28 (F(5,20) = 6.07, *p* = 0.001). A post hoc *t*-test revealed significant differences between the baseline and PID1 (*p* = 0.002) and between baseline and PID28 (*p* = 0.001). The SSIT group data are presented in Figure 1C. We found that, at any time point, the PPI ratios were affected when the broadband noise (top panels, Figure 1C) and 12–14 kHz frequency band (Figure 1C, bottom right panel) were used. However, the GAP ratio at 12–14 kHz was significantly increased at PID7 and PID28. These results resemble trends in previous reports wherein the 10–20 kHz frequency band caused the most tinnitus after overdosing on sodium salicylate [20].

**Figure 2:**
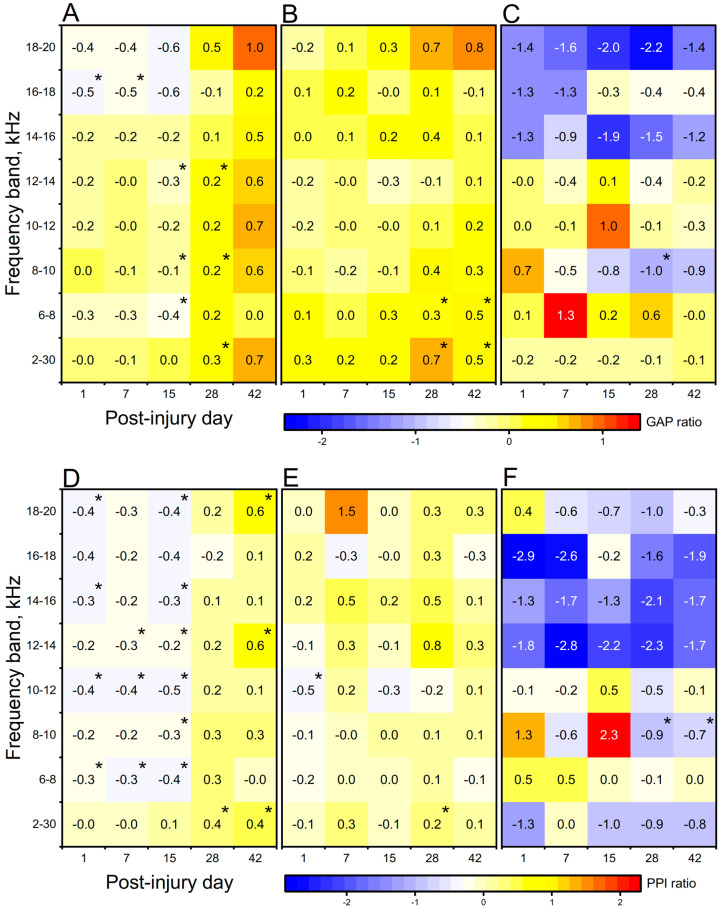
The results of acoustic startle response GAP (**A**–**C**) and PPI (**D**–**F**) evaluation in SSIT (**A**,**D**), sham (**B**,**E**), and BLAST (**C**,**F**) cohorts. The presented time-series data are average responses with subtracted baseline prescreening values. Statistical significance is indicated with asterisk (*, *p* < 0.05). The negative values indicate the decreasing response compared to baseline. Large inhibitory trend is evident in high-frequency bands in animals exposed to a single blast in both tests. This effect is relatively weak, and most of the time-frequency band groups are above the statistical significance threshold.

Exposure to blast and high-intensity impulsive noise can cause both conductive and sensorineural damage to the auditory system. We designed our experiments considering this possibility, and thus, the ears of animals were protected by earplugs, which minimizes the probability of conductive damage. This approach also allows us to examine the sensorineural changes in CAS that are solely caused by exposure to a shock wave (BLAST cohort) or high-intensity noise (sham cohort). What is also noticeable is that some BLAST and sham animals have larger than 1 GAP/PPI ratios when compared with the results from previous studies, either after a long time of noise exposure [33] or blast exposure [15]. A ratio larger than one is the indicator of hyperacusis, which is also the co-morbidity of blast-induced hearing abnormality. In order to visualize the PPI/GAP ratio results with respect to time, in Figure 2, we found no ratio more than 1 in the SSIT group, while in both BLAST and sham groups, the hyperacusis is revealed at PID-7 and PID-15. These results indicate both the blast and noise exposure-induced hyperacusis but the blast caused more severe hyperacusis.

### 3.2. Blast Increases Thresholds of Auditory Brainstem Responses 

**Figure 3:**
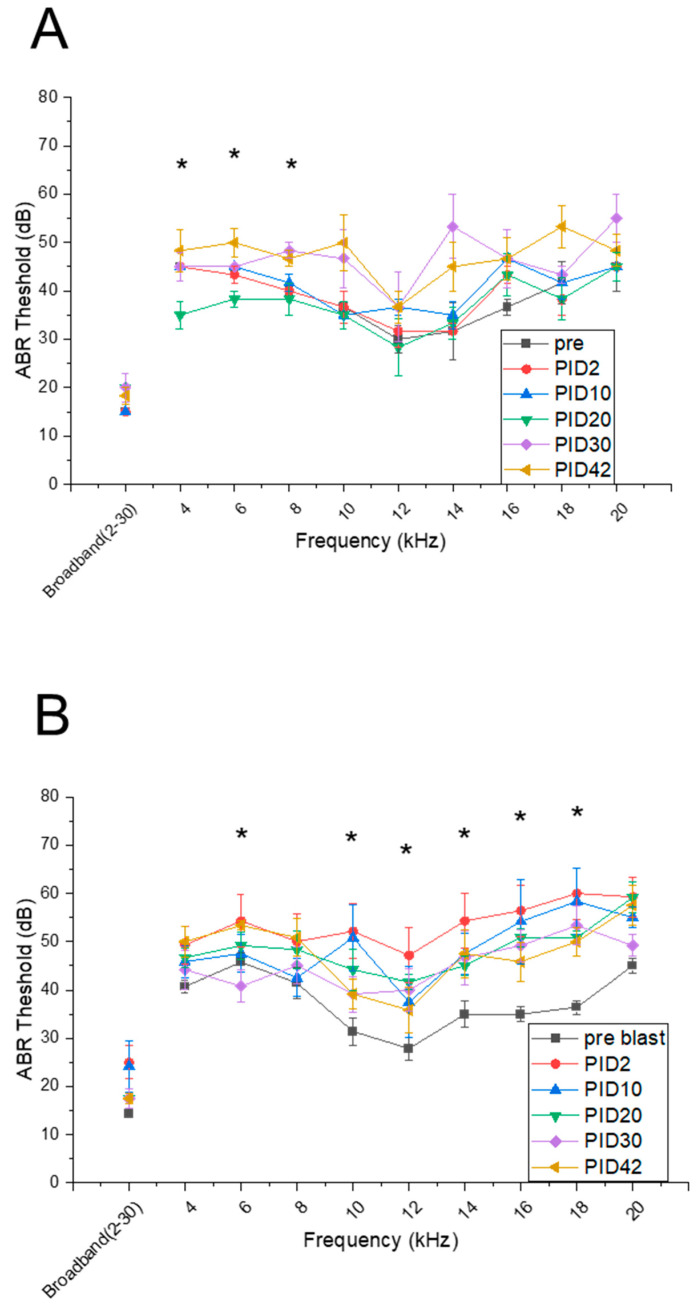

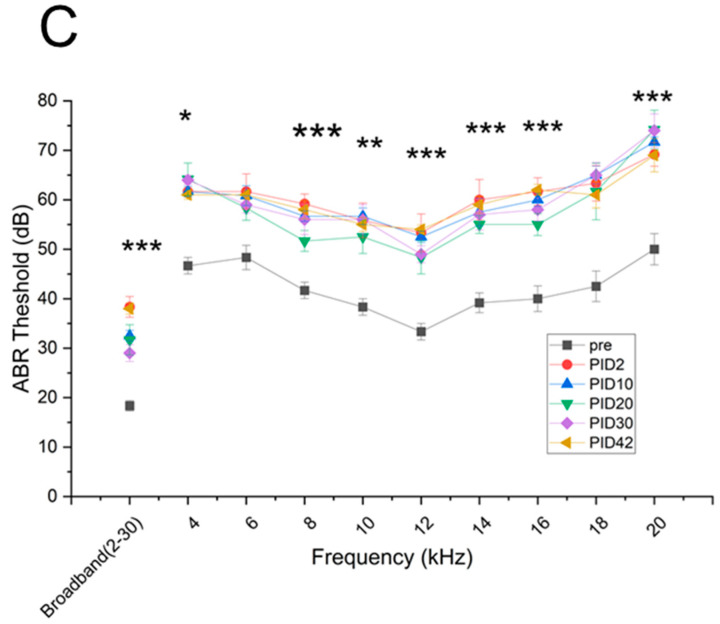
The auditory brainstem response thresholds after: (**A**). noise exposure (sham), (**B**). 180 kPa blast, and (**C**). continuous sodium salicylate injection. Both cohorts of animals subjected to noise and blast exposure had their ears protected by earplugs to avoid tympanic membrane rupture. Repeated measure ANOVA was used for the statistical analysis of behavioral tests, with the following significance thresholds: * *p* < 0.05, ** *p* < 0.005, and *** *p* < 0.0005.

The ABR thresholds for different test groups are shown in Figure 3. The sham group results indicate there was no significant change compared with baseline and PID2 (Figure 3A). At other combinations of time points and frequencies used in our experimental design, there are no clear trends with relatively large variability among the data (20–60 dB range). Only in a few instances was there a departure from the baseline (marked as PRE below). Specifically, the ABR thresholds have increased at lower frequencies of 4, 6, 8 kHz: (1) 4 kHz: PID20-PID40 (*p* = 0.013), PID20-PID (*p* = 0.048) and PID20-PID30 (*p* = 0.025), (2) 6 kHz: PRE-PID42 (*p* = 0.028), PID2-PID10 (*p* = 0.040), PID2-PID42 (*p* = 0.004), PID20-PID42 (*p* = 0.006), PID20-PID30 (*p* = 0.046), (3) 8 kHz: PID2-PID30 (*p* = 0.005), and PID2-PID42 (*p* = 0.001). 

In the cohort of animals exposed to blast compared with baseline, the threshold generally increased by 10–20 dB, particularly at the higher frequencies (>14 kHz, Figure 2B). In general, the blast exposure resulted in acute threshold shift at PID2, which then subsided to a certain extent but was sustained in the chronic phase post-injury (PID30 and 42). The statistically significant ABR threshold shifts at various frequencies are as follows: (1) 6 kHz: PRE-PID42 (*p* = 0.02s0), PID30-PID42 (*p* = 0.007), (2) 10 kHz: PRE-PID2 (*p* = 0.007), (3) 12 kHz: PRE-PID20 (*p* = 0.001), (4) 1 s 4 kHz: PRE-PID2 (*p* = 0.010), PRE-PID20 (*p* = 0.017), (5) 16 kHz: PRE-PID2 (*p* = 0.002) PRE-PID20 (*p* = 0.001), PRE-PID30 (*p* = 0.003), (6) 18 kHz: PRE-PID2 (*p* = 0.001), PRE-PID10 (*p* = 0.009), PRE-PID20 (*p* = 0.001), PRE-PID30 (*p* = 0.002), PRE-PID42 (*p* = 0.002), (7) 20 kHz: PRE-PID2 (*p* = 0.007), PRE-PID10 (*p* = 0.003).

The ABR threshold was also increased in the SSIT group, and these data are the easiest to interpret since there is a very distinct separation between the baseline (denoted as PRE in Figure 2C) and results collected after the salicylate injections. In this group of animals, we observed the threshold shift after the broadband noise exposure (2–32 kHz), which was not identified in the other two cohorts of animals. A 10+ dB threshold shift increase suggests that the bridge circuit from the cochlear to inferior colliculus was compromised by sodium salicylate injection. Our results are consistent with those previously reported [18].

**Figure 4:**
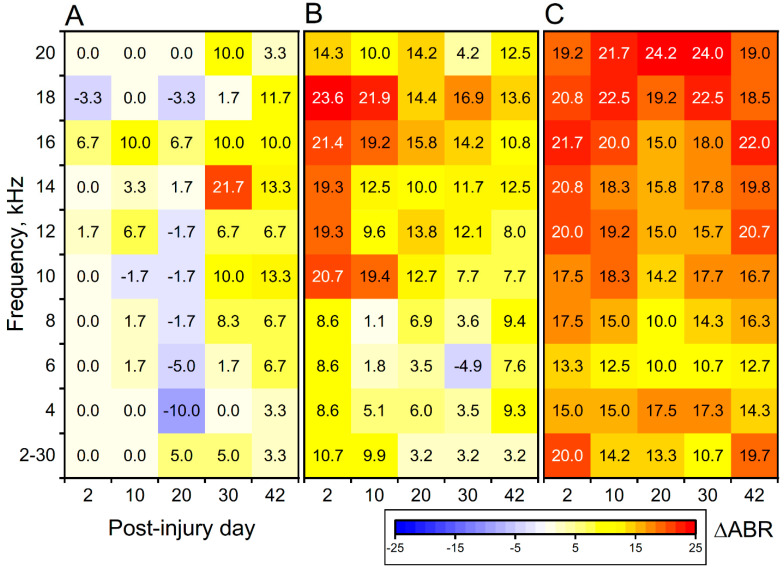
The auditory brainstem response thresholds after: (**A**). noise exposure (sham), (**B**). 180 kPa blast, and (**C**). Continuous sodium salicylate injection. The heatmap of differential ABR threshold values (treatment group data with subtracted baseline data, i.e., those taken before the treatment) are presented. Both cohorts of animals subjected to noise and blast exposure had their ears protected by earplugs to avoid tympanic membrane rupture.

In Figure 4, we calculated and plotted ABR threshold changes for each post-time point compared with pre-conditions. The results show that the noise exposure condition was mostly recoverable, while both blast and SSIT groups showed the injury persisted with greater severity. 

### 3.3. Blast Effect on Neurotransmitter Receptors and Synaptic Proteins in Auditory Cortex

**Figure 5:**
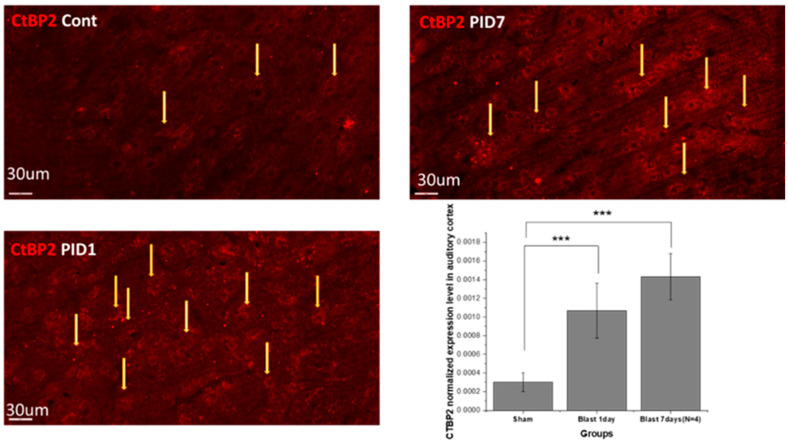
CtBP2 immunohistochemistry staining images and normalized CtBP2 expression in auditory cortex according to immunofluorescence intensity. CtBP, Carboxyl terminal binding protein. The yellow arrows indicate the samples of the positive stains(the cells number are not counted instead the normalized fluorescence intensity is quantified and shown in the graph. Repeated measure ANOVA was used for the statistical analysis of behavioral tests, with the following significance thresholds: *** *p* < 0.0005.

Carboxyl terminal binding protein (CtBP2) expression. The normalized CtBP2 expression characterized by the intensity of the immunohistochemistry is shown in Figure 5. Compared with the sham group, the expressions of CtBP2 at post-blast day 1 and day 7 were both significantly increased. 

**Figure 6:**
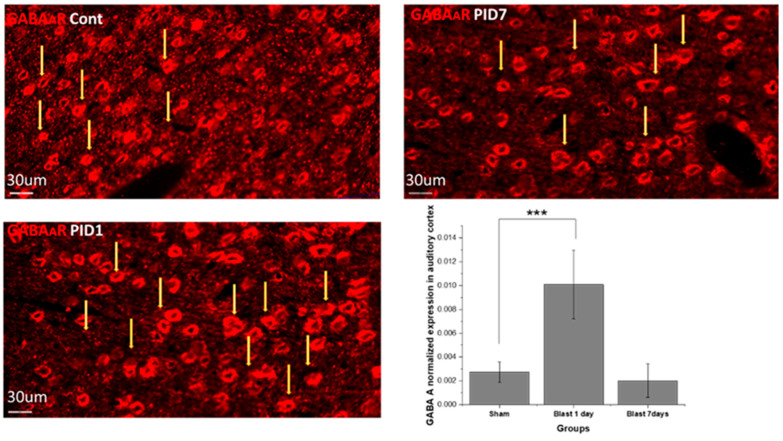
GABAAR immunohistochemistry staining images and normalized GABAAR expression in auditory cortex according to immunofluorescence intensity. CtBP, Carboxyl terminal binding protein. The yellow arrows indicate the samples of the positive stains(the cells number are not counted instead the normalized fluorescence intensity is quantified and shown in the graph. Repeated measure ANOVA was used for the statistical analysis of behavioral tests, with the following significance thresholds: *** *p* < 0.0005.

**Figure 7:**
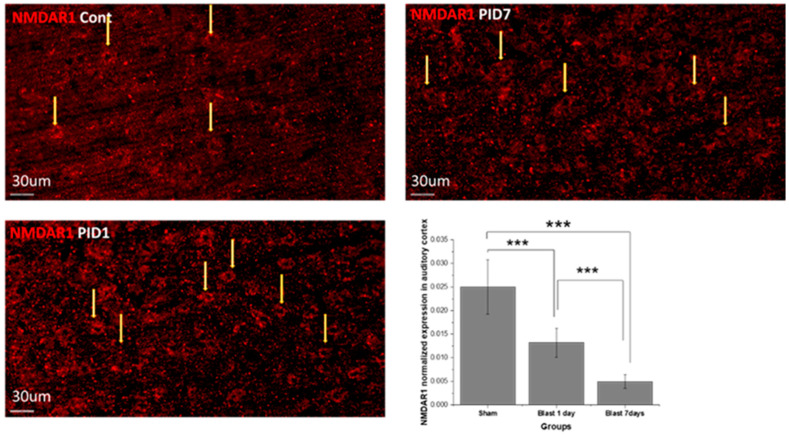
NMDAR1 immunohistochemistry staining images and normalized NMDAR1 expression in auditory cortex according to immunofluorescence intensity. CtBP, Carboxyl terminal binding protein. The yellow arrows indicate the samples of the positive stains(the cells number are not counted instead the normalized fluorescence intensity is quantified and shown in the graph. Repeated measure ANOVA was used for the statistical analysis of behavioral tests, with the following significance thresholds: *** *p* < 0.0005.

Expression of neurotransmitter receptors: GABRA1 and NMDAR1. Normalized GABRA1 expression is shown in Figure 6. The expression observed over 7 days showed the unimodal trend: at post-blast day 1, the expression was significantly upregulated compared with that of the sham (*p* = 3.1 × 10^−4^); while at day 7, the expression was restored to the low level as the sham. The normalized NMDAR1 is shown in Figure 7, showing the consistent decreasing trend throughout 7 days after blast injury: compared with sham on day 1 (*p* = 9.9 × 10^−4^); day 7 (*p* = 1.2 × 10^−5^); compared with day 1 and day 7, *p* = 3.4 × 10^−4^.

### 3.4. Increased Anxiety Revealed by EPM

**Figure 8:**
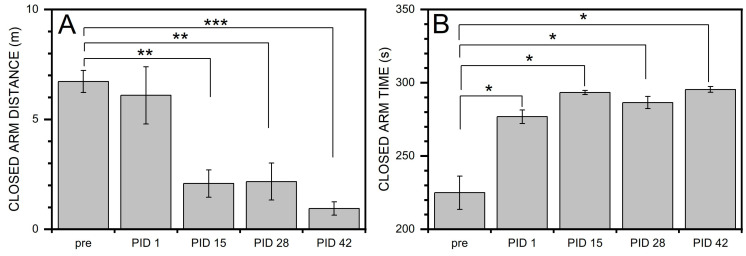

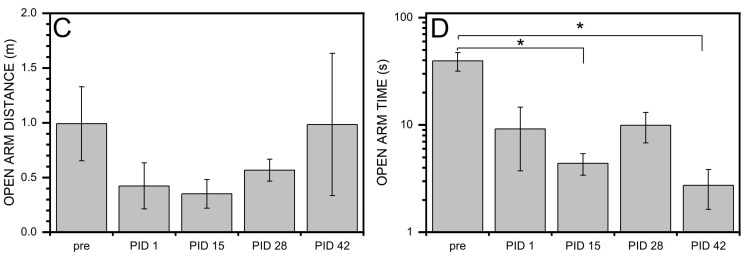
Summary of EPM after sodium salicylate injection. (**A**) Closed arm distance; (**B**) Closed arm time; (**C**) Open arm distance; and (**D**) Open arm time. Repeated measure ANOVA was used for the statistical analysis of behavioral tests, with the following significance thresholds: * *p* < 0.05, ** *p* < 0.005, and *** *p* < 0.0005.

The data presented in Figure 8 revealed that continuous sodium salicylate overdosing leads to a dramatic decrease (3 to 6 times) of closed-arm distance traveled after two or more weeks (PID15 to 42) compared with baseline: (1) PID15 (*p* = 1.7 × 10^−4^), (2) PID28 (*p* = 3.2 × 10^−4^), and (3) PID42 (*p* = 6.4 × 10^−4^). At PID42 compared with PID1, *p* = 0.007. Simultaneously, animals required more time to complete this task at all four-time points in our experimental design compared to baseline: PID1 (*p* = 0.002), PID15 (*p* = 1.4 × 10^−4^), PID28 (*p* = 0.001), and PID42 (*p* = 3.6 × 10^−4^). The trends observed in the open-arm task are not as dramatic, as illustrated by the lack of changes in the open-arm distance task. However, the time to complete the open-arm task compared with the baseline was significantly reduced at PID15 (*p* = 0.001) and PID42 (*p* = 0.002).

**Figure 9:**
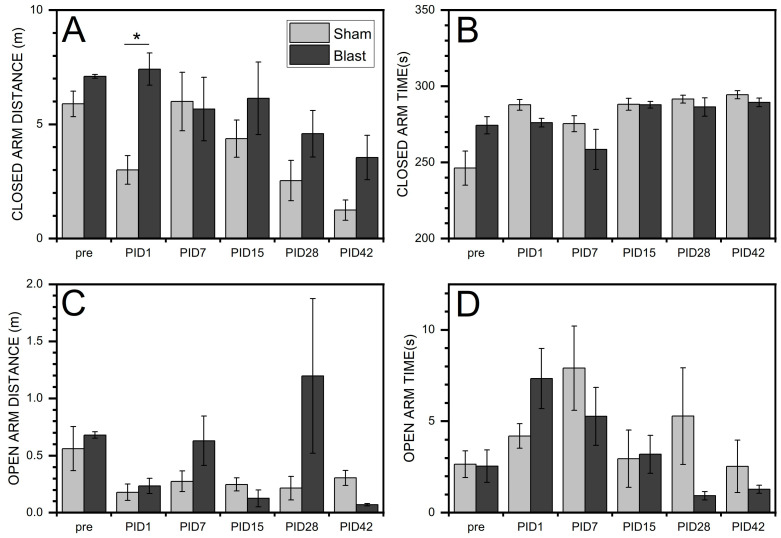
Summary of EPM among sham and blast conditions: significant increase of the closed arm traveled distance in the BLAST group compared with sham group. (**A**) Closed arm distance; (**B**) Closed arm time; (**C**) Open arm distance; and (**D**) Open arm time. Repeated measure ANOVA was used for the statistical analysis of behavioral tests, with the following significance thresholds: * *p* < 0.05.

In Figure 9, the trends in open/close-arm distance traveled and time tasks are presented for both blast and sham groups. In the blast group, at PID1, a significant change was shown (*p* = 0.029) when compared with sham. These data indicate that both noise and blast can lead to increased anxiety and vigilance, as shown by increased exploratory and locomotive intent. 

### 3.5. Altered Sleep Pattern after Blast 

**Figure 10:**
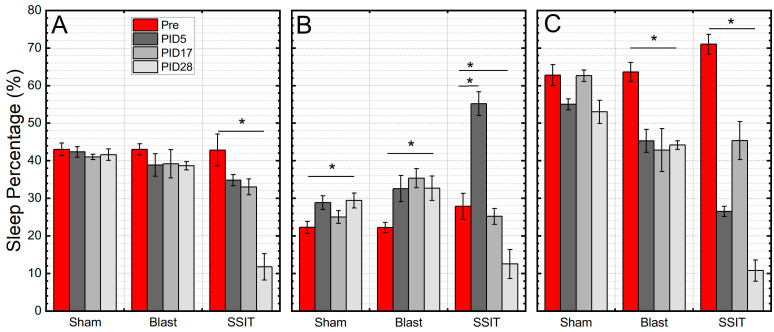
Summary of total sleep percentage (**A**), sleep percentage in the dark (**B**) and in the light (**C**) for three test groups used in the study. Repeated measure ANOVA was used for the statistical analysis of behavioral tests, with the following significance thresholds: * *p* < 0.05.

After continuous sodium salicylate injection, the sleep study was implemented at PIDs 5, 17, and 28. Sleep percentage in the light, sleep percentage in the dark, and total sleep percentage (the percentage of sleep both in the dark and light) were recorded. In Figure 10A, we present the temporal changes in sleep percentage and how sleep partitioning into light and dark phases was affected by SS administration. We compared these data with the baseline sleep percentage before the SS injection. The total sleep percentage was reduced progressively throughout 28 days. Day 28 data were significantly decreased compared with baseline (*p* = 8.6 × 10^−4^), day 5 (*p* = 1.3 × 10^−4^), and day 17 (*p* = 5.5 × 10^−4^). Sleep percentage in the light decreased, while sleep percentage in the dark was significantly increased at day 5. For sleep percentage in the dark: at day 5, the percentage was significantly increased compared with baseline (*p* = 1.7 × 10^−4^); at day 17 and 28, the percentage was significantly decreased (*p* = 1.30 × 10^−5^, *p* = 2.7 × 10^−5^, respectively). For day sleep percentage, at days 5, 17, and 28, the sleep percentages were significantly reduced compared with baseline (*p* = 3.7 × 10^−8^, *p* = 0.001, *p* = 3.7 × 10^−7^, respectively). In sum, the SSIT group showed an acute increase in night sleep coupled with decreased day sleep.

Our results demonstrate that, compared with sham, blast causes increased sleep in the dark (Figure 10B) and reduced sleep in the light (Figure 10C), while the whole sleep percentage was not significantly changed (Figure 10A). For sleep percentage in the light, at day 17 and 28 there was a significant difference compared sham with blast (*p* = 0.017, *p* = 0.043). For in the dark sleep, at day 17, there was a significant difference compared with sham (*p* = 0.008). 

## 4. Discussion

This study demonstrates that blast exposure can preferentially induce tinnitus in the absence of any damage to the peripheral auditory system (PAS), especially at 180 kPa peak overpressure. A sustained increase of ABR thresholds in both blast-exposed and sodium salicylate overdosing groups indicates the induction of disrupted neuronal circuitry between the peripheral auditory system (PAS) and central auditory system (CAS). However, a single exposure to blast trauma showed less severe and sustained outcomes than chronic sodium salicylate administration. In CAS, sustained increased expression of ribbon synapse marker CtBP2 indicates the inflammatory response in the auditory cortex. However, the excitatory and inhibitory neurotransmitter receptor levels (GABRA1 and NMDAR1) showed acute imbalances. These findings strongly suggested that blast-induced tinnitus involves both PAS and CAS abnormalities.

### 4.1. Tinnitus-like Symptom and Hyperacusis after Blast

As noted in the Results Section, we observed significant changes in ASR responses in both blast-exposed and sodium salicylate overdosing groups. These results reinforce that blast waves induced more tinnitus-like symptoms than complete hearing loss when controlling for conductive damage using earplugs. This study also further demonstrated the occurrence of hyperacusis in both an acute time frame and high frequency ranges. It was also found that, at broadband frequency, there was minimal hearing loss, but there was the significant perception of tinnitus, demonstrating that, with the protection of earplugs, blast-exposed rats are more likely to develop tinnitus. The prior literature focused on studying [33,34,35] continuous (a few hours around 100 dB) sound exposure for tinnitus development. In our study, the noise generated by the shock wave was short (within a few milliseconds) but louder (192 dB). Therefore, it was reiterated that the effect of the duration of noise weighed more than the sound overpressure level for tinnitus induction. However, the correlation and mechanism of hyperacusis and tinnitus are still open to future investigations. In this study, we also observed great subject differences in ASR diagnosis. This also coincided with both animal studies and field observations. According to previous studies, it was found that only part of the group developed tinnitus after a blast in a rat animal model [15]. Among soldiers, considering the variety of blast dosage with respect to each individual, 59% of military personnel with the confirmed diagnosis of deployment-related mild TBI had tinnitus. Both suggested that tinnitus development was subject-dependent, and it can vary even when exposed to the same blast.

### 4.2. Shock Wave Caused Severe and Sustained Functional Injury in the Neuronal Circuit between Cochlea and Inferior Colliculus in High-Frequency Processing

ABR evaluated the functional connection between PAS and CAS. The ABR is a frequently used method to record auditory evoked potentials generated along the pathway from the inner ear auditory nerves to the inferior colliculus in the midbrain [36,37]. The normal ABR consists of five prominent waves that occur during the first 10 ms after the presentation of a transient sound stimulus. We examined the auditory brainstem response threshold in our three groups. Our data showed that blast injury caused more severe but recoverable effects on threshold shifts compared with the SSIT group throughout the whole frequency span and more severe at high frequencies. This observation can be explained by the anatomy of the cochlea. The threshold elevation of the ABR after blast exposure was primarily caused by outer hair cell dysfunction induced by stereociliary bundle disruption [10]. According to the topographical frequency mapping of the cochlea, the stereociliary bundles at the front entrance of the cochlea are for high-frequency mapping, which is under the highest influence of the shock wave. Another study [38] found that, in both humans and chinchillas, intracochlear pressures increased with applied sound pressure. In addition, more detailed morphological changes in the cochlear sensory hairs were examined by means of a scanning electron microscope, showing sensory hairs of the basal and the second turns were damaged more markedly than other turns, which corresponds to a frequency larger than 5 kHz [39].

The temporal pattern of ABR thresholds for the blast group showed signs of recovery even though the injury persisted at the last time point we examined (day 42). On the other hand, there was no significant long-term change in the sham group. Here, we revealed the different pathophysiology of blast-induced and single short noise-induced hearing distortion. Due to the limitation of the time points in this study, the chronic comparison is still open to investigation.

### 4.3. Acute Astrocytic Response and Metabolic Changes after Blast 

Zou et al. indicated that the expression of CtBP2 was associated with astrocyte activation and proliferation in rat CNS after TBI. In fact, increased astrocyte reactivity was one of the hallmarks of consequences of TBI in both post-mortem [40] and rodent [41] studies. In auditory brain regions, Kallakuri et al. [16] identified the chronic (one month after 22 psi blast injury) astrocyte elevation in the auditory cortex. Moreover, CtBP2 can respond to NADH/NAD+ ratio levels [42]. In clinical studies of TBI patients, the cellular NADH/NAD+ redox state changed, followed by abnormal downstream energy metabolism [43]. All of them indicated that the acute CtBP2 upregulation was the upstream event after the blast, which then led to astrocyte proliferation and metabolic disorders.

### 4.4. Neurotransmitter Receptor Alters Acute Imbalance in Blast TBI 

GABA A receptor α1 participates in opening Cl^−^ channels and leads to hyperpolarization of the post-synaptic cell with other subunits, while α1 and γ2 are involved in phasic inhibition. Phasic inhibition decreases the hyperexcitability of the post-synaptic cell and plays an important role in the creation and modulation of theta and gamma oscillations [34]. These oscillations are critical in maintaining neural functions. Recent studies also showed these oscillations as the biomarkers of major mental illness, including depression [35,36]. Our data showed increased GABA A receptor level at post-blast day 1, which suggested that the cortex region has compensatory changes in response to the increased neural traffic from the ascending auditory pathway. In the sodium salicylate-injected animal, GABAergic inhibition is overall decreased in the auditory cortex. This suggests different mechanisms of hearing impairment concerning drug and blast-induced effects. The NMDA receptor (NMDAR) has the role of customized synaptic plasticity and long-term potentiation and potentially contributes to diseases such as schizophrenia and Alzheimer’s disease. Within all subunits, NMDAR1 binds glycine, responsible for receptor deactivation. The functional NMDAR1 is essential for the activity of the NMDAR receptor [44]. Our results show the strong, persistent compromised NMDAR1, which leads to NMDAR malfunction or even neural death. 

Furthermore, we demonstrated that, on a cellular level, the shock wave exerted more severe damage to the neuronal auditory system when compared to a single shot of loud noise. Blast exposure has been well known to cause damage to fluid and air-occupied PAS, including ruptured TM and hair cell loss. Damage to hair cells in the cochlea can create vascular, metabolic, and chemical changes to normal cell signaling, which can accordingly disrupt the afferent signaling in CAS. Moreover, sensorineural hearing loss (SNHL) is the prevalent type of auditory impairment in blast trauma [7]. Here, we found the imbalanced neurotransmitter receptor expression level in the auditory cortex, favoring the bottom-up mechanism: CAS responds to the ascending signals accordingly. That is, the received signals change from PAS caused the plasticity of the CAS changes to counteract the disrupted afferent signals. The combination of increased inhibition and decreased incitation led to the cortex’s suppression as the mechanism of self-protection in response of abnormal ascending neuronal signals. 

### 4.5. Chronic Sleep Deficits and Increased Anxiety after Blast

All the above-mentioned sensorineural damage concerning both PAS and CAS coexisted with adverse behavior traits. Tinnitus is often associated with the development of anxiety and sleep deficits. In follow-up studies of veterans, there are complaints about sleep disturbances, including changes in sleep quality and quantity [45], which further leads to increased anxiety. For the sleep pattern study in our animal model, we did not identify the overall sleep loss; however, we found a decrease in dark sleep and an increase in light sleep percentage. Rats, as nocturnal animals, normally spend more time sleeping during the day (the light phase) and are more active at night (the dark phase). The shift in sleep patterns in this study indicated that even a single exposure to loud noise or a shock wave may lead to prolonged disturbance in sleep. These changes are, however, not as pronounced as those observed in the chronic tinnitus model (SSIT cohort). The findings in our animal study also corresponded with the typical symptom of blast-induced TBI: sleep and wake disturbances. The clinical data of blast TBI patients showed that sleep problems mediate the effect of a positive TBI screening on the development of mental health disorders, and sleep problems are an early indicator of risk for post-traumatic stress disorder (PTSD) or depression [45,46].

The sleep pattern shifts only happened at sub-acute and chronic time points, which indicates that the sleep disorder took time to develop but would sustain. Moreover, the initiating of the sleep pattern shift occurred at the same time as the increased anxiety level we observed by EPM. According to the time points we examined here, there was no conclusive information about the relationship between anxiety disorder and sleep pattern disruptions. 

## 5. Conclusions

Our study suggested that blast with the earplugs protection compromised normal auditory processing functions with an intact auditory conduction system. Our 180 kPa blast trauma caused the tinnitus-like symptoms in our rat animal model, which correlated with neuronal damage and adverse behavioral outcomes. In the CAS, we observed overall inhibitory effects in the auditory cortex, which is the protection response in response to the abnormally increased ascending neuronal signals from PAS. For the injury, a blast can cause two auditory hazards: shock wave and noise. Our data suggested that shock wave, compared with noise, played a significant role in tinnitus induction and neuronal damage. Shock wave and noise exposure incited different responses that may require distinct preventive interventions. It is important to stress that a single exposure is an unlikely scenario in any military operation, and thus, multiple-exposure models are necessary to capture the occupational exposure conditions. Future investigations should elucidate mechanical loading on fluids as well as the air-filled auditory system and the corresponding biological/biochemical damages using the multiple exposure injury paradigm. Of importance should also be the employment of electrophysiology of auditory neurons, to examine structure–function relationships. Our pathophysiological study incorporated both PAS and CAS in the rat animal model, providing guidance to the occupational protection gear design. By studying the temporal changes, we can better understand how this morbidity progresses, subsides, or persists, which can give the healthcare provider more reference for determining the regimen of medical interference. 

## Data Availability

Data is unavailable due to privacy or ethical restrictions.
